# 2-[1-(1-Phenyl­eth­yl)imidazolidin-2-yl­idene]malononitrile

**DOI:** 10.1107/S1600536812010823

**Published:** 2012-03-28

**Authors:** Xiao-Wei Liu, Liang-Zhong Xu

**Affiliations:** a College of Chemistry and Molecular Engineering, Qingdao University of Science and Technology, Qingdao 266042, People’s Republic of China

## Abstract

In the title compound, C_14_H_14_N_4_, the imidazolidine moiety is nearly planar, having an N—C—N—C torsion angle of 4.43 (3)°. The crystal structure is characterized by classical N—H⋯N hydrogen bonds, which form inversion dimers.

## Related literature

For the biological activity of compounds containing a 2-(imidazolidin-2-ylidene)malononitrile group, see: Hense *et al.* (2002 ▶). For a related structure, see: Feng *et al.* (2008 ▶). For the synthesis of the title compound, see: Jeschke *et al.* (2002 ▶).
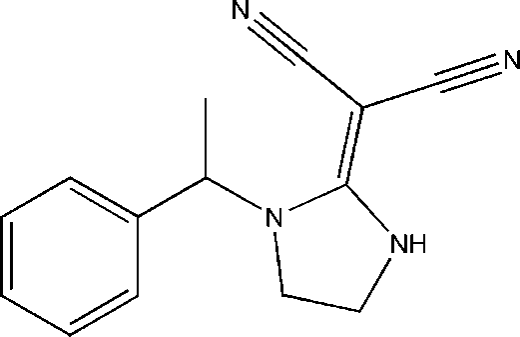


## Experimental

### 

#### Crystal data

C_14_H_14_N_4_
*M* *_r_* = 238.29
Triclinic, 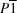

*a* = 6.6446 (13) Å
*b* = 8.0106 (16) Å
*c* = 12.847 (3) Å
α = 90.51 (3)°
β = 101.85 (3)°
γ = 107.76 (3)°
*V* = 635.5 (3) Å^3^
*Z* = 2
Mo *K*α radiation
μ = 0.08 mm^−1^
*T* = 295 K
0.46 × 0.41 × 0.11 mm

#### Data collection

Rigaku R-AXIS RAPID IP diffractometer
Absorption correction: multi-scan (*CrystalClear*; Rigaku, 2005 ▶) *T*_min_ = 0.965, *T*_max_ = 0.992
6293 measured reflections
2898 independent reflections
2112 reflections with *I* > 2σ(*I*)
*R*_int_ = 0.019

#### Refinement

*R*[*F* ^2^ > 2σ(*F* ^2^)] = 0.044
*wR*(*F* ^2^) = 0.145
*S* = 1.15
2898 reflections
164 parameters
H-atom parameters constrained
Δρ_max_ = 0.21 e Å^−3^
Δρ_min_ = −0.19 e Å^−3^

### 

Data collection: *CrystalClear* (Rigaku, 2005 ▶); cell refinement: *CrystalClear*; data reduction: *CrystalClear*; program(s) used to solve structure: *SHELXS97* (Sheldrick, 2008 ▶); program(s) used to refine structure: *SHELXL97* (Sheldrick, 2008 ▶); molecular graphics: *SHELXTL* (Sheldrick, 2008 ▶); software used to prepare material for publication: *SHELXTL*.

## Supplementary Material

Crystal structure: contains datablock(s) I, global. DOI: 10.1107/S1600536812010823/rk2338sup1.cif

Structure factors: contains datablock(s) I. DOI: 10.1107/S1600536812010823/rk2338Isup2.hkl

Supplementary material file. DOI: 10.1107/S1600536812010823/rk2338Isup3.cml

Additional supplementary materials:  crystallographic information; 3D view; checkCIF report

## Figures and Tables

**Table 1:** Hydrogen-bond geometry (Å, °)

*D*—H⋯*A*	*D*—H	H⋯*A*	*D*⋯*A*	*D*—H⋯*A*
N2—H2*A*⋯N4^i^	0.86	2.27	3.032 (2)	148

Symmetry code: (i) 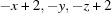.

**Fig. 1.**
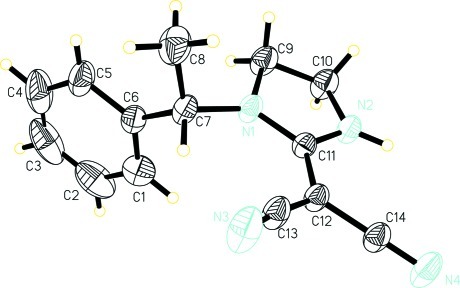
View of the title compound with the atom numbering scheme. Displacement ellipsoids are drawn at the 50% probability level. H atoms are presented as a small spheres of arbitrary radius.

## supplementary crystallographic information

### Comment

Recently, imidazolidin is an important kind of group in organic chemistry. Compounds containing the 2-(imidazolidin-2-ylidene)malononitrile group have attracted much interest because compounds containing a imidazole ring system are well known as efficient insecticide in pesticides, and have good plant-growth regulatory activity for a wide variety of crops (Hense, *et al.*, 2002). We report herein the crystal structure of title compound.

In title molecule (Fig. 1), the bond lengths and angles of the imidazolidin rings are in agreement with those in previous reports (Feng *et al.*, 2008). The imidazolidin moiety has a small torsion angle N1–C11–N2–C10 = 4.43 (3)° which is nearly closed to a plane. The main plane of imidazolidin ring and the benzene ring make a dihedral angle of 87.18 (2)°. The crystal structure is characterized by N2–H2A···N4^i^ classical intermolecular hydrogen bonds and centosymmetrical dimers with using these. H-bonds parameters: N2–H2A = 0.86Å, H2A···N4^i^ = 2.27Å, N2···N4^i^ = 3.032 (2)Å and angle N2–H2A···N4^i^ = 147.6°. Symmetry code: (i) -*x*+2, -*y*, -*z*+2.

### Experimental

The title compound was prepared according Jeschke *et al.*, 2002. Single crystals suitable for X-ray measurement were obtained by recrystallization from the mixture of acetone and methanol at room temperature.

### Refinement

H atoms were placed in calculated positions, with C–H = 0.93-0.98Å and N–H = 0.86Å, and included in the final cycles of refinement using a riding model, with *U*_iso_(H) = 1.5*U*_eq_(C) for methyl H atoms and 1.2*U*_eq_(C,N) for other.

### Crystal data

**Table:**

C_14_H_14_N_4_	*Z* = 2
*M**_r_* = 238.29	*F*(000) = 252
Triclinic, *P*1	*D*_x_ = 1.245 Mg m^−^^3^
Hall symbol: -P 1	Mo *K*α radiation, λ = 0.71073 Å
*a* = 6.6446 (13) Å	Cell parameters from 4704 reflections
*b* = 8.0106 (16) Å	θ = 6.1–55.0°
*c* = 12.847 (3) Å	µ = 0.08 mm^−^^1^
α = 90.51 (3)°	*T* = 295 K
β = 101.85 (3)°	Block, colourless
γ = 107.76 (3)°	0.46 × 0.41 × 0.11 mm
*V* = 635.5 (3) Å^3^	

### Data collection

**Table:**

Rigaku R-AXIS RAPID IP diffractometer	2898 independent reflections
Radiation source: Rotating Anode	2112 reflections with *I* > 2σ(*I*)
Graphite monochromator	*R*_int_ = 0.019
φ and ω scans	θ_max_ = 27.5°, θ_min_ = 3.0°
Absorption correction: multi-scan (*CrystalClear*; Rigaku, 2005)	*h* = −8→8
*T*_min_ = 0.965, *T*_max_ = 0.992	*k* = −10→10
6293 measured reflections	*l* = −16→16

### Refinement

**Table:**

Refinement on *F*^2^	Secondary atom site location: difference Fourier map
Least-squares matrix: full	Hydrogen site location: inferred from neighbouring sites
*R*[*F*^2^ > 2σ(*F*^2^)] = 0.044	H-atom parameters constrained
*wR*(*F*^2^) = 0.145	*w* = 1/[σ^2^(*F*_o_^2^) + (0.063*P*)^2^ + 0.0913*P*] where *P* = (*F*_o_^2^ + 2*F*_c_^2^)/3
*S* = 1.15	(Δ/σ)_max_ < 0.001
2898 reflections	Δρ_max_ = 0.21 e Å^−^^3^
164 parameters	Δρ_min_ = −0.19 e Å^−^^3^
0 restraints	Extinction correction: *SHELXL97* (Sheldrick, 2008), Fc^*^=kFc[1+0.001xFc^2^λ^3^/sin(2θ)]^-1/4^
Primary atom site location: structure-invariant direct methods	Extinction coefficient: 0.210 (17)

### Special details

**Table:**

Geometry. All s.u.'s (except the s.u.' in the dihedral angle between two l.s. planes) are estimated using the full covariance matrix. The cell s.u.'s are taken into account individually in the estimation of s.u.'s in distances, angles and torsion angles; correlations between s.u.'s in cell parameters are only used when they are defined by crystal symmetry. An approximate (isotropic) treatment of cell s.u.'s is used for estimating s.u.'s involving l.s. planes.
Refinement. Refinement of *F*^2^ against ALL reflections. The weighted *R*-factor *wR* and goodness of fit *S* are based on *F*^2^, conventional *R*-factors *R* are based on *F*, with *F* set to zero for negative *F*^2^. The threshold expression of *F*^2^ > σ(*F*^2^) is used only for calculating *R*-factors(gt) *etc*. and is not relevant to the choice of reflections for refinement. *R*-factors based on *F*^2^ are statistically about twice as large as those based on *F*, and *R*-factors based on ALL data will be even larger.

### Fractional atomic coordinates and isotropic or equivalent isotropic displacement parameters (Å^2^)

**Table:**

	*x*	*y*	*z*	*U*_iso_*/*U*_eq_	
N1	0.43363 (19)	0.16514 (14)	0.79468 (10)	0.0520 (3)	
N2	0.72640 (19)	0.19909 (15)	0.91821 (10)	0.0536 (3)	
H2A	0.8196	0.1712	0.9659	0.064*	
N3	0.1806 (3)	−0.35508 (19)	0.77933 (15)	0.0898 (6)	
N4	0.8269 (3)	−0.20051 (18)	0.96591 (14)	0.0808 (5)	
C1	0.4326 (3)	0.1738 (3)	0.56429 (16)	0.0782 (5)	
H1A	0.5353	0.1251	0.5998	0.094*	
C2	0.4528 (5)	0.2430 (4)	0.4674 (2)	0.1102 (9)	
H2B	0.5692	0.2406	0.4384	0.132*	
C3	0.3038 (7)	0.3147 (3)	0.41400 (19)	0.1218 (12)	
H3A	0.3166	0.3591	0.3482	0.146*	
C4	0.1366 (5)	0.3208 (3)	0.45755 (19)	0.1070 (9)	
H4A	0.0360	0.3716	0.4219	0.128*	
C5	0.1144 (3)	0.2526 (2)	0.55405 (15)	0.0762 (5)	
H5A	−0.0013	0.2578	0.5828	0.091*	
C6	0.2612 (2)	0.17651 (18)	0.60873 (12)	0.0535 (4)	
C7	0.2351 (2)	0.09009 (18)	0.71185 (12)	0.0521 (4)	
H7A	0.2186	−0.0345	0.6984	0.063*	
C8	0.0397 (3)	0.0987 (3)	0.75267 (16)	0.0819 (6)	
H8A	0.0358	0.0410	0.8177	0.123*	
H8B	−0.0899	0.0411	0.7003	0.123*	
H8C	0.0495	0.2194	0.7658	0.123*	
C9	0.5134 (3)	0.35393 (18)	0.82776 (14)	0.0637 (4)	
H9A	0.4198	0.3861	0.8676	0.076*	
H9B	0.5236	0.4237	0.7666	0.076*	
C10	0.7356 (3)	0.3781 (2)	0.89770 (15)	0.0652 (5)	
H10A	0.8496	0.4324	0.8606	0.078*	
H10B	0.7582	0.4487	0.9633	0.078*	
C11	0.5563 (2)	0.08185 (16)	0.85453 (10)	0.0427 (3)	
C12	0.5237 (2)	−0.10079 (17)	0.85565 (11)	0.0463 (3)	
C13	0.3310 (3)	−0.23639 (18)	0.81102 (13)	0.0571 (4)	
C14	0.6899 (2)	−0.15619 (17)	0.91645 (12)	0.0544 (4)	

### Atomic displacement parameters (Å^2^)

**Table:**

	*U*^11^	*U*^22^	*U*^33^	*U*^12^	*U*^13^	*U*^23^
N1	0.0518 (7)	0.0364 (6)	0.0600 (7)	0.0146 (5)	−0.0060 (5)	0.0044 (5)
N2	0.0505 (7)	0.0411 (6)	0.0607 (7)	0.0139 (5)	−0.0058 (5)	0.0029 (5)
N3	0.0747 (10)	0.0488 (8)	0.1153 (13)	0.0034 (7)	−0.0228 (9)	0.0179 (8)
N4	0.0751 (10)	0.0476 (7)	0.1022 (11)	0.0227 (7)	−0.0245 (8)	0.0060 (7)
C1	0.0797 (12)	0.0727 (11)	0.0818 (12)	0.0196 (10)	0.0231 (10)	0.0037 (9)
C2	0.138 (2)	0.0916 (17)	0.0893 (16)	−0.0008 (16)	0.0555 (16)	−0.0052 (14)
C3	0.193 (3)	0.0721 (14)	0.0601 (12)	−0.0100 (18)	0.0169 (18)	0.0092 (11)
C4	0.146 (2)	0.0751 (14)	0.0712 (13)	0.0233 (14)	−0.0239 (15)	0.0214 (11)
C5	0.0804 (12)	0.0661 (10)	0.0737 (11)	0.0273 (9)	−0.0090 (9)	0.0156 (9)
C6	0.0550 (8)	0.0407 (7)	0.0575 (8)	0.0128 (6)	−0.0004 (6)	0.0030 (6)
C7	0.0453 (7)	0.0438 (7)	0.0607 (8)	0.0137 (6)	−0.0025 (6)	0.0071 (6)
C8	0.0537 (10)	0.1076 (16)	0.0791 (12)	0.0189 (10)	0.0124 (8)	0.0145 (11)
C9	0.0651 (10)	0.0386 (7)	0.0789 (10)	0.0170 (7)	−0.0038 (8)	0.0042 (7)
C10	0.0620 (9)	0.0405 (7)	0.0816 (11)	0.0122 (7)	−0.0036 (8)	0.0037 (7)
C11	0.0420 (6)	0.0404 (6)	0.0454 (7)	0.0135 (5)	0.0080 (5)	0.0068 (5)
C12	0.0472 (7)	0.0380 (6)	0.0505 (7)	0.0140 (5)	0.0028 (5)	0.0086 (5)
C13	0.0573 (9)	0.0399 (7)	0.0659 (9)	0.0141 (6)	−0.0029 (7)	0.0128 (6)
C14	0.0563 (8)	0.0363 (7)	0.0627 (9)	0.0132 (6)	−0.0024 (7)	0.0064 (6)

### Geometric parameters (Å, º)

**Table:**

N1—C11	1.3356 (16)	C5—C6	1.381 (2)
N1—C9	1.4682 (18)	C5—H5A	0.9300
N1—C7	1.4702 (18)	C6—C7	1.517 (2)
N2—C11	1.3371 (18)	C7—C8	1.516 (2)
N2—C10	1.4452 (19)	C7—H7A	0.9800
N2—H2A	0.8600	C8—H8A	0.9600
N3—C13	1.147 (2)	C8—H8B	0.9600
N4—C14	1.1496 (19)	C8—H8C	0.9600
C1—C6	1.380 (3)	C9—C10	1.517 (2)
C1—C2	1.382 (3)	C9—H9A	0.9700
C1—H1A	0.9300	C9—H9B	0.9700
C2—C3	1.362 (4)	C10—H10A	0.9700
C2—H2B	0.9300	C10—H10B	0.9700
C3—C4	1.355 (4)	C11—C12	1.4129 (18)
C3—H3A	0.9300	C12—C14	1.4062 (19)
C4—C5	1.377 (3)	C12—C13	1.410 (2)
C4—H4A	0.9300		
			
C11—N1—C9	110.32 (12)	C8—C7—H7A	107.2
C11—N1—C7	128.66 (11)	C6—C7—H7A	107.2
C9—N1—C7	120.86 (11)	C7—C8—H8A	109.5
C11—N2—C10	112.21 (12)	C7—C8—H8B	109.5
C11—N2—H2A	123.9	H8A—C8—H8B	109.5
C10—N2—H2A	123.9	C7—C8—H8C	109.5
C6—C1—C2	120.6 (2)	H8A—C8—H8C	109.5
C6—C1—H1A	119.7	H8B—C8—H8C	109.5
C2—C1—H1A	119.7	N1—C9—C10	103.10 (12)
C3—C2—C1	120.6 (3)	N1—C9—H9A	111.1
C3—C2—H2B	119.7	C10—C9—H9A	111.1
C1—C2—H2B	119.7	N1—C9—H9B	111.1
C4—C3—C2	119.5 (2)	C10—C9—H9B	111.1
C4—C3—H3A	120.3	H9A—C9—H9B	109.1
C2—C3—H3A	120.3	N2—C10—C9	102.14 (12)
C3—C4—C5	120.6 (2)	N2—C10—H10A	111.3
C3—C4—H4A	119.7	C9—C10—H10A	111.3
C5—C4—H4A	119.7	N2—C10—H10B	111.3
C4—C5—C6	121.0 (2)	C9—C10—H10B	111.3
C4—C5—H5A	119.5	H10A—C10—H10B	109.2
C6—C5—H5A	119.5	N1—C11—N2	109.74 (11)
C1—C6—C5	117.79 (17)	N1—C11—C12	128.42 (12)
C1—C6—C7	119.56 (14)	N2—C11—C12	121.84 (12)
C5—C6—C7	122.59 (16)	C14—C12—C13	115.29 (12)
N1—C7—C8	110.06 (13)	C14—C12—C11	117.82 (12)
N1—C7—C6	109.75 (12)	C13—C12—C11	126.52 (12)
C8—C7—C6	115.10 (13)	N3—C13—C12	174.86 (15)
N1—C7—H7A	107.2	N4—C14—C12	179.51 (18)
			
C6—C1—C2—C3	−0.1 (3)	C5—C6—C7—C8	−2.0 (2)
C1—C2—C3—C4	1.2 (4)	C11—N1—C9—C10	13.82 (18)
C2—C3—C4—C5	−1.1 (4)	C7—N1—C9—C10	−170.38 (14)
C3—C4—C5—C6	0.0 (3)	C11—N2—C10—C9	12.63 (19)
C2—C1—C6—C5	−1.0 (3)	N1—C9—C10—N2	−15.03 (18)
C2—C1—C6—C7	176.36 (17)	C9—N1—C11—N2	−6.47 (17)
C4—C5—C6—C1	1.0 (3)	C7—N1—C11—N2	178.16 (14)
C4—C5—C6—C7	−176.22 (17)	C9—N1—C11—C12	173.57 (15)
C11—N1—C7—C8	106.76 (18)	C7—N1—C11—C12	−1.8 (2)
C9—N1—C7—C8	−68.19 (19)	C10—N2—C11—N1	−4.43 (18)
C11—N1—C7—C6	−125.59 (15)	C10—N2—C11—C12	175.54 (14)
C9—N1—C7—C6	59.46 (18)	N1—C11—C12—C14	172.25 (14)
C1—C6—C7—N1	56.03 (18)	N2—C11—C12—C14	−7.7 (2)
C5—C6—C7—N1	−126.78 (16)	N1—C11—C12—C13	−15.1 (2)
C1—C6—C7—C8	−179.18 (16)	N2—C11—C12—C13	164.94 (15)

### Hydrogen-bond geometry (Å, º)

**Table:**

*D*—H···*A*	*D*—H	H···*A*	*D*···*A*	*D*—H···*A*
N2—H2*A*···N4^i^	0.86	2.27	3.032 (2)	148

Symmetry code: (i) −*x*+2, −*y*, −*z*+2.
